# Benefits of Continuous Outpatient Orthopedic Consultations for Both Upper and Lower Body on the Mortality Rates of Hemodialysis Patients

**DOI:** 10.7759/cureus.75576

**Published:** 2024-12-12

**Authors:** Kazuhiko Yoshikawa, Takuya Kishi, Ayako Takamori, Taku Goto, koji Onozawa, Kuniyasu Takagi, Fumihiro Kuroki, Yuichiro Akiyoshi, Takuaki Yamamoto

**Affiliations:** 1 Orthopedic Surgery, International University of Health and Welfare Graduate School of Medicine, Okawa, JPN; 2 Cardiology, Department of Graduate School of Medicine, International University of Health and Welfare, Okawa, JPN; 3 Clinical Research Center, Saga University Hospital, Saga, JPN; 4 Emergency, Kouhou-kai Takagi Hospital, Okawa, JPN; 5 Nephrology, Kouhou-kai Takagi Hospital, Okawa, JPN; 6 Internal Medicine, International University of Health and Welfare Graduate School of Medicine, Okawa, JPN; 7 Orthopedic Surgery, Kouhou-kai Takagi Hospital, Okawa, JPN; 8 Orthopedic Surgery, Fukuoka University, Fukuoka, JPN

**Keywords:** consultation, hemodialysis, mortality, orthopedics, regional general hospital

## Abstract

Background: The purpose of the present observational study was to examine whether there is a difference in prognosis for hemodialysis patients with or without continued orthopedic outpatient visits over five years.

Methods: One hundred and thirteen hemodialysis patients who visited the dialysis center of Takagi Hospital, Okawa, Japan, as of December 2017 were included in this study. Data were collected from the medical records until December 2022. All 113 patients were divided into two groups: patients who continuously visited the orthopedic outpatient department (n = 59) and those who did not (n = 54). Patients who had orthopedic consultation were divided into three semi-groups: patients who consulted for the upper body (n = 11), the lower body (n = 22), and both the upper and lower bodies (n = 26).

Results: During the five-year follow-up period, 13 out of 59 patients (22.0%) who had orthopedic consultation died, and this ratio tended to be lower compared to the mortality rate of patients without orthopedic consultation (37.0%) but not significant (P < 0.08). Duration of hemodialysis was significantly longer in patients with orthopedic consultation (P = 0.009). The mortality rate was significantly lower in patients who consulted for both upper and lower bodies than those without orthopedic consultation (P < 0.05, respectively). These differences were not observed in patients who consulted for only upper or lower bodies.

Conclusion: The mortality of hemodialysis patients was significantly lower in the group which was undertaking continuous outpatient orthopedic consultations for both upper and lower bodies, which suggested that periodical consultation with orthopedics might be critical for hemodialysis patients.

## Introduction

Maintenance hemodialysis is applied for patients with end-stage renal failure worldwide [1-3]. The prognosis of renal failure patients with hemodialysis is regulated by several factors, including the stage of renal failure, patient comorbidities, and patient conditions, including cerebral and cardiovascular disorders, gastrointestinal disorders, and malnutrition [4-7]. Our previous studies indicated that frailty, gastrointestinal disorders, and significant cardiovascular events might increase the risk of shortening the life of hemodialysis patients [8,9].

Several reviews and reports indicate that the risk of bone fracture is exacerbated during maintenance hemodialysis [10-13]. A recent cohort study suggested that aging, female gender, lower albumin, and higher vascular calcification score were independently associated with an increased risk of bone fracture during hemodialysis, and bone fracture risk was diminished by active vitamin D therapy [12]. Few studies focus on the influence of bone fractures on the life prognosis of hemodialysis patients. In contrast, one reports that hemodialysis patients' mortality and hospitalization rates significantly increased in the first month after bone fracture [13]. In this regard, orthopedic surgeons' continued outpatient prevention, detection, and treatment of fractures in hemodialysis patients may improve the long-term prognosis of dialysis patients. However, no reports examine the relationship between outpatient orthopedic visits and mortality in dialysis patients. 

Considering these backgrounds, we hypothesized that access to an orthopedic surgeon for hemodialysis patients would impact prognosis by enabling the detection, treatment, and prevention of fractures. The purpose of the present observational study was to examine whether there is a difference in prognosis for hemodialysis patients with or without continued orthopedic outpatient visits over five years.

## Materials and methods

**Figure 1:**
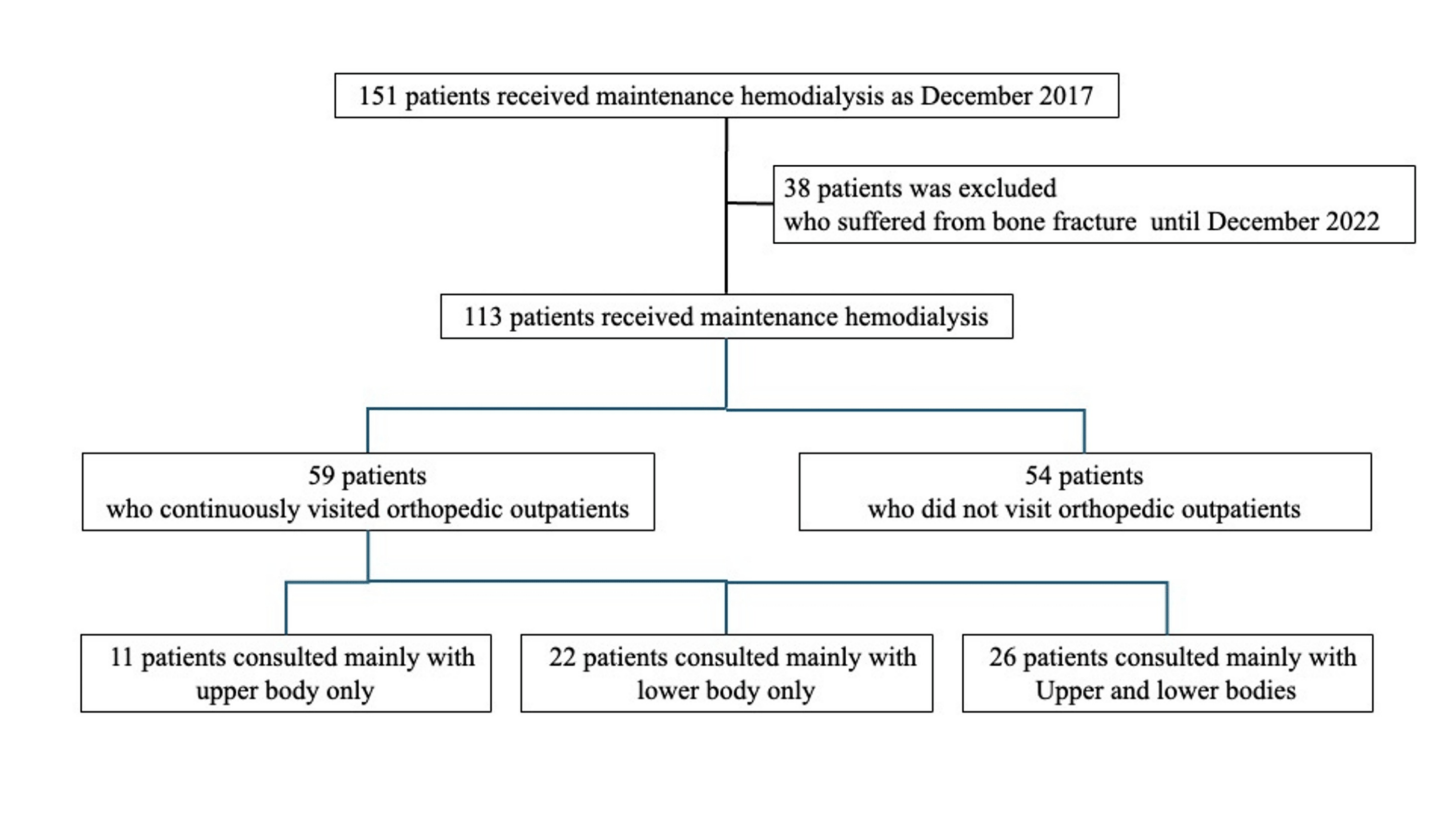
Flowchart of the present study One hundred fifty-one patients received maintenance hemodialysis for renal failure at the hemodialysis center of nephrology in the Takagi Hospital as of December 2017, and after 38 patients who suffered from bone fractures from the follow-up period until December 2022 were excluded, 113 patients with hemodialysis were included in the present study. They were divided into two groups: patients who continuously visited orthopedic outpatients (n = 59) and patients who did not visit the orthopedic outpatient clinic (n = 54). In addition, patients who continuously visited orthopedic outpatients were divided into three semi-groups: patients consulted mainly for the upper body only (n = 11), lower body only (n = 22), and for both upper and lower bodies (n = 26).

One hundred fifty-one patients received maintenance hemodialysis for renal failure at the Hemodialysis Center of Nephrology in the Takagi Hospital, Okawa, Japan, as of December 2017 (Figure 1). The present study excluded 38 patients who suffered from bone fractures from the follow-up period until December 2022 (Figure 1). Finally, 113 patients with hemodialysis were included in the present study, and 113 patients were divided into two groups: patients who continuously visited orthopedic outpatients of the Takagi Hospital for chronic musculoskeletal issues or preemptive visits (n = 59) and patients who did not visit orthopedic outpatients (n = 54) (Figure 1). In addition, patients who continuously visited the orthopedic outpatient clinic were divided into three semi-groups: patients consulted mainly for the upper body only (n = 11), lower body only (n = 22), and both upper and lower bodies (n = 26) (Figure 1).

Data on patient characteristics and event incidences, including death, were collected in the electronic medical record for five years until December 2022. Gender, age, duration of hemodialysis, the origin of renal failure, mortality during the follow-up period, gastrointestinal bleeding, and medication during hemodialysis were recorded. A patient's blood test in December 2017 was examined as follows: hemoglobin, total protein, cholesterol, serum calcium, phosphorus, parathyroid hormone, and iron. This study was conducted by the Declaration of Helsinki, with ethical review and approval from the Takagi Hospital's Ethical Committee (Kouhou-kai Ethical Committee #495) and the International University of Health and Welfare Ethical Committee (21-Ifh-027).

Baseline characteristics of group patients were compared using chi-square, Fisher's exact test for categorical variables, and Student's t-test for continuous variables. Cary, NC, USA) was used for all analyses, and statistical significance was defined as P < 0.05. 

## Results

As indicated in Table 1, 59 out of 113 patients (52.2%) with maintenance hemodialysis for renal failure regularly visited the orthopedic outpatient office of Takagi Hospital. Our hospital has been actively consulting orthopedic surgeons to check for fractures and assess and treat fracture risk in hemodialysis patients. An orthopedic surgeon checks all fracture detection, as there have been cases where fractures have been missed by non-orthopedic surgeons. Since the introduction of dialysis, we have been proactive in ensuring that patients are regularly and permanently seen by an orthopedic surgeon at least once a year. All patients consulted with or visited the orthopedics department in our hospital. Compared to patients without orthopedic consultations, the mortality rate during the five-year follow-up period in patients with consultation tended to be lower (13/59: 22.0% vs. 20/54: 37.0%, P = 0.08) but insignificant. Gender distribution, age, origin of renal failure, and gastrointestinal bleeding were not different for patients with or without orthopedic consultation. 

**Table 1:** Patient characteristics of chronic hemodialysis patients with or without orthopedic consultation (total number of patients = 113) Nephrogenic diseases, including chronic nephritis and nephrosclerosis; Data are mean ± SD. * = significant difference (P<0.05); DM: diabetes mellitus

Parameters	Consultation (+)	Consultation (-)	P-value
	n = 59	n = 54	
Male/female	38 / 21	35 / 19	0.96
Age (year)	66.6 ± 11.3	66.1 ± 15.0	0.85
Body mass index (kg/m^2^)	22.8 ± 3.1	21.9 ± 2.8	0.18
Smoking	4 (7 %)	5 (9 %)	0.13
Drinking habit	18 (31 %)	15 (28 %)	0.09
History of bone fracture	2 (3%)	3 (6%)	0.07
Peritoneal dialysis	1 (2%)	2 (4%)	0.11
Origin of renal failure			
Diabetes mellitus	22	17	0.13
Nephrogenic diseases	36	37	0.47
Gastrointestinal bleeding	10	11	0.67
Mortality during the five-year follow-up period	13/59 (22.0%）	20/54 (37.0%)	0.08

In Table 2, patients who had orthopedic outpatient consultation were divided into three semi-groups: regular, continuous orthopedic consultation for chronic musculoskeletal issues or preemptive visits mainly with upper body (humerus, forearm, wrist, and forearm) only (n = 11), lower body (lumbar spine, sacrum, femoral neck, femoral inter-trochanteric, femoral sub-trochanteric, and ankle) only (n = 22), and both upper and lower body (n = 26). Orthopedic assessment of the upper and lower body was carried out by examination and radiographic evaluation. Among the patients, lower body bone fractures at the lumber spine and sacrum were treated conservatively with rest and conservative treatment. Femoral neck and femoral inter- or subtrochanteric fractures were operated on. Ankle fractures were treated with cast immobilization. Compared to patients without orthopedic consultation, mortality during the five-year follow-up period was significantly improved in patients consulted with upper and lower bodies (P < 0.05). This improvement in mortality was not observed in patients consulted with upper body only or lower body only. Regarding gender distribution, patients consulted with the upper body were limited to males, and the other two semi-groups of lower only and both upper and lower were not different compared to no consultation patients. Age was not different among the three consultation semi-groups and no consultation group. The origin of hemodialysis was not different among the evaluated groups. 

**Table 2:** Classification of orthopedic consultation patients under hemodialysis with body site to be examined: upper body (n = 11), lower body (n = 22), or upper + lower body (n = 26). Data are mean ± SD; ** = P < 0.05 compared to the value of no consultation; DM: diabetes mellitus

Parameters	Upper body	Lower body	Upper + lower body	No consultation
	n = 11	n = 22	n = 26	n = 54
Male/female	11 / 0*	9 / 13	12 / 14	35 / 19
Age (years)	65.0 ± 7.0	70.1 ± 11.1	64.3 ±12.5	66.1 ± 15.0
Origin of renal failures				
DM	6	7	9	17
Others	5	15	16	37
Mortality during the five-year follow-up period	4/11 (36.4%)	7/22 (31.8%)	2/26 (7.7%)*	20/54 (37.0%)

Table 3 shows the medication for patients with hemodialysis, and there was no significant difference between with or without orthopedic consultation. Orthostatic consultation means an orthostatic outpatient office for preemptive visits or chronic musculoskeletal issues. There were no patients with calcium-sensing receptor medication. Regarding a blood test at the entry period of December 2017 (Table 4), evaluated factors including hemoglobin, total protein, cholesterol, serum calcium, serum phosphate, parathyroid hormone, and serum iron were not different between the two groups.

**Table 3:** Medication for patients who received maintenance hemodialysis: comparison between with or without orthopedic consultation NSAIDs: non-steroidal anti-inflammatory drugs; ACE: angiotensin-converting enzyme; Ca: calcium

Parameters	Consultation (+)	Consultation (-)	P value
	n = 59	n = 54	
Anticoagulants	6	5	0.8
Aspirin	21	12	0.08
Antiplatelets except for aspirin	8	10	0.55
Erythropoiesis stimulants	50	46	0.52
Iron preparations	33	23	0.09
Proton pump inhibitors	26	16	0.07
Histamine-2 receptor blockers	15	14	0.87
NSAIDs contentious use	3	3	0.21
Adrenocorticosteroid	3	3	0.96
Vitamin D receptor activator	41	44	0.3
ACE inhibitors	12	11	0.61
Ca channel blockers	33	29	0.27

**Table 4:** Blood test results of hemodialysis patients on December 2017 Data are mean ± SD.

Parameters	Consultation (+)	Consultation (-)	P value
	n = 59	n = 54	
Hemoglobin (g/dL)	11.1 ± 1.4	10.9 ± 1.4	0.42
Total protein (g/dL)	6.4 ± 0.6	6.3 ± 0.6	0.61
Cholesterol (mg/dL)	152 ± 39	148 ± 35	0.53
Corrected serum calcium (mg/dL)	8.9 ± 0.6	9.1 ± 0.5	0.17
Serum phosphorus (mg/dL)	5.9 ± 1.6	5.4 ± 1.6	0.06
Intact parathyroid hormone (pg/mL)	166 ± 146	146 ± 112	0.42
Serum iron (mg/dL)	65.2 ± 27.0	61.3 ± 28.6	0.47

## Discussion

The present retrospective analysis indicated the following: 1) mortality of hemodialysis patients who had orthopedic consultation tended to be lower compared to those who did not opt for orthopedic consultation (not significant); 2) mortality was significantly lower in hemodialysis patients who had orthopedic consultation for both upper and lower bodies compared to those who did not have an orthopedic consultation; and 3) mortality was not significantly different between hemodialysis patients who had orthopedic consultation for upper or lower bodies only compared to those who did not have orthopedic consultation. However, we calculated that the sufficient sample size was 163 cases by type 1 error (0.05), type 2 error (0.80), and effect size (0.5) with two tails on the Cochran-Mantel-Haenszel test and considered that the present sample size (total 113 cases) could not avoid type 1 and 2 errors. Although further clinical study should be necessary, our present results indicated that periodical consultation with orthopedics for both upper and lower bodies might be critical for hemodialysis patients.

The most important result of this study is the finding that mortality was significantly lower in the hemodialysis patients with orthopedic consultation regarding both the upper and lower body than in those without orthopedic consultation. Patients with hemodialysis have a high risk of bone fracture [10-13], and aging, female gender, lower albumin, and a higher vascular calcification score were independently associated with an increased risk of bone fracture during hemodialysis [12]. On the other hand, greater selectivity in prescribing psychoactive drugs and more efficient treatment of secondary hyperparathyroidism can reduce the risk of hip and other bone fractures in hemodialysis patients [14]. The oral charcoal adsorbent AST-120 and renin-angiotensin system inhibitors may improve bone quality and decrease fracture risk in chronic kidney disease patients [15]. In this study, orthopedic consultation visits are also meant to provide medical management of metabolic bone disease and secondary or tertiary hyperparathyroidism. Reducing fracture risk in chronic kidney disease patients on dialysis requires individualized strategies, as fracture prevention in the general population may not apply to them. However, no previous clinical study demonstrated the benefits of orthopedic consultation in patients with hemodialysis.

Moreover, it needs to be clarified whether fracture prevention improves the prognosis of hemodialysis patients. The logistics of shifting for orthopedic consultation may pose a significant challenge for severely ill patients undergoing hemodialysis. In this study, orthopedic surgeons visited hemodialysis patients in the dialysis unit during dialysis when they could not visit an orthopedic outpatient clinic for mobility reasons. The results of this study demonstrate the importance of first making the attending physician aware of the need for orthopedic visits in hemodialysis patients. In this context, the results of this study are significant in the management of dialysis patients because they show that consultation with an orthopedic surgeon for upper and lower body fractures and the risk of fracture in dialysis patients may improve their prognosis.

The present study could not test the cause of the lack of a significant difference in mortality in hemodialysis patients who had an upper or lower body-only consultation with an orthopedic surgeon compared to those without an orthopedic consultation. Nephrologists who manage hemodialysis patients may consult orthopedic surgeons regarding fractures that occur or are discovered incidentally, but only for the upper or lower body. Considering the results of this study, consultation with an orthopedic surgeon for systemic fracture risk assessment and fracture prevention may be necessary for the management of dialysis patients.

In this study, it was impossible to verify what interventions and changes in bone density or frailty occurred due to the consultation with the orthopedic surgeon. In our research, the orthopedic surgeon consulted and worked with the primary care physician to provide surgical or conservative treatment if a fracture was found. The surgeon introduced outpatient rehabilitation in addition to assessing the risk of fracture and advising on pharmacological treatment. Therefore, further research is needed to determine which specific factors can be addressed by consultation and intervention with an orthopedic surgeon to improve the prognosis of hemodialysis patients.

There were several study limitations. First, the present study, with a limited number of patients, was a single-center and retrospective analysis, and bias could not be denied because the data were collected from medical records and not entirely engraved in all cases. Second, the observation period was five years, which might be evaluated for a more extended period. Third, the dialysis conditions in the groups with and without orthopedic consultation have yet to be adequately assessed. Fourth, we did not fully consider the detailed clinical observations, including a site of gastrointestinal bleeding, nutrition, frailty, infection, and muscle mass.

Moreover, the majority of dialysis-dependent patients have diabetes. Diabetic neuropathy, retinopathy, Charcot foot/ankle complications, very high cardiovascular risk, and advanced peripheral vascular disease are prevalent in this patient population. These limitations warrant further explorations shortly. 

## Conclusions

Although the data collected in this single-center retrospective study are insufficient and the causal relationship has not been thoroughly examined, mortality might be significantly lower in the hemodialysis patients with orthopedic consultation regarding both the upper and lower body than in those without orthopedic consultation. In the management of hemodialysis patients, consultation with an orthopedic surgeon for upper and lower body fractures and the risk of fracture in hemodialysis patients may decrease the mortality. Prevention, detection, and treatment of fractures throughout the body from an orthopedic surgeon's perspective may benefit hemodialysis patients' prognosis.

## References

[1] Kawanishi H, Akiba T, Masakane I (2009). Standard on microbiological management of fluids for hemodialysis and related therapies by the Japanese Society for Dialysis Therapy 2008. Ther Apher Dial.

[2] Agrawaal KK (2022). Maintenance hemodialysis among patients visiting nephrology unit in a tertiary care center: a descriptive cross-sectional study. JNMA J Nepal Med Assoc.

[3] Shibata M (2023). Safety management of dialysis fluid in Japan: important duties and responsibilities of clinical engineers. Blood Purif.

[4] Huang CH, Chao JY, Ling TC (2023). Effect of dialysis modalities on risk of hospitalization for gastrointestinal bleeding. Sci Rep.

[5] Okamura M, Inoue T, Ogawa M (2022). Rehabilitation Nutrition in patients with chronic kidney disease and cachexia. Nutrients.

[6] Okuno S (2021). Significance of adipose tissue maintenance in patients undergoing hemodialysis. Nutrients.

[7] Tsuneyoshi S, Matsukuma Y, Kawai Y (2021). Association between geriatric nutritional risk index and stroke risk in hemodialysis patients: 10-years outcome of the Q-Cohort study. Atherosclerosis.

[8] Kitajima A, Kishi T, Yamanouchi K (2023). A retrospective analysis of risk factors for mortality during hemodialysis at a general hospital that treats comprehensive diseases. Intern Med.

[9] Kishi T, Kitajima A, Yamanouchi K (2022). Low body mass index without malnutrition is an independent risk factor for major cardiovascular events in patients with hemodialysis. Int Heart J.

[10] Yoshida M, Nakashima A, Doi S, Maeda K, Ishiuchi N, Naito T, Masaki T (2021). Lower geriatric nutritional risk index (GNRI) is associated with a higher risk of fractures in patients undergoing hemodialysis. Nutrients.

[11] Komaba H, Ketteler M, Cunningham J, Fukagawa M (2021). Old and new drugs for the management of bone disorders in CKD. Calcif Tissue Int.

[12] Matias PJ, Laranjinha I, Azevedo A (2020). Bone fracture risk factors in prevalent hemodialysis patients. J Bone Miner Metab.

[13] Tentori F, McCullough K, Kilpatrick RD, Bradbury BD, Robinson BM, Kerr PG, Pisoni RL (2014). High rates of death and hospitalization follow bone fracture among hemodialysis patients. Kidney Int.

[14] Jadoul M, Albert JM, Akiba T (2006). Incidence and risk factors for hip or other bone fractures among hemodialysis patients in the Dialysis Outcomes and Practice Patterns Study. Kidney Int.

[15] Yamamoto S, Fukagawa M (2017). Uremic toxicity and bone in CKD. J Nephrol.

